# Complete Genome Sequence of *Alteromonas stellipolaris* LMG 21856, a Budding Brown Pigment-Producing Oligotrophic Bacterium Isolated from the Southern Ocean

**DOI:** 10.1128/genomeA.00137-16

**Published:** 2016-03-24

**Authors:** Jigang Chen, Xing Wang, Sidong Zhu, Yong Chen, Jifang Yang

**Affiliations:** College of Biological and Environmental Sciences, Zhejiang Wanli University, Ningbo, China

## Abstract

Here, we report the complete genome sequence of *Alteromonas stellipolaris* LMG 21856, which was isolated from seawater collected from the Southern Ocean. *A. stellipolaris* LMG 21856 is a budding, psychrotrophic, brown pigment-producing, and oligotrophic bacterium*.* The complete genome of this bacterium contains 4,686,200 bp, with a G+C content of 43.6%.

## GENOME ANNOUNCEMENT

*Alteromonas* is a genus of *Proteobacteria* found in the open ocean and in coastal seawater and currently contains 14 species. The genome sequences of >20 strains of the genus *Alteromonas* have been reported or released to public databases, revealing a number of features that are related to the adaptation to their habitats (1–8). The species described here, *Alteromonas stellipolaris* LMG 21856, was isolated from Southern Ocean seawater after an enrichment technique was conducted in dialysis chambers (9). Here, we report the genome sequence of this extremophile, which is a budding, psychrotrophic, brown pigment-producing, and oligotrophic bacterium belonging to the *Gammaproteobacteria* (10).

*A. stellipolaris* LMG 21856 was cultured in Zobell 2216E at 20°C (10). Genomic DNA was prepared using a DNA extraction kit (BioTech), according to the manufacturer’s instructions. Whole-genome shotgun DNA sequencing of *A. stellipolaris* LMG 21856 was performed at the Beijing Genomics Institute (BGI, Shenzhen, China) using Solexa paired-end sequencing technology. A total of 493 Mb high-quality reads with approximately 105-fold coverage of the entire genome were generated. All reads were assembled into 33 contigs and 21 scaffolds using SOAP*denovo* (http://soap.genomics.org.cn/soapdenovo.html). PCR amplification and Sanger sequencing were performed to close all gaps. The annotation was conducted using combined results from RAST (11) and Glimmer version 3.0 (12). tRNAs and rRNAs were identified using the tRNAscan-SE (13), RNAmmer (14), and Rfam (15) databases, and the contigs were searched against the NCBI NR, Swiss-Prot, COG, TrEMBL, InterProScan, and KEGG protein databases to annotate the gene descriptions.

The completed genome has 4,686,200 bases and is composed of 3,995 predicted coding sequences, with an average G+C content of 43.6%. Fifty-eight tRNA genes and 15 rRNA genes were detected in the complete genome. Most protein-coding sequences were related to the metabolism of amino acids and derivatives and carbohydrates. Genes associated with stress response and protein metabolism were also abundant. Function-based comparison with metabolic construction of the three previously published genomes of *Alteromonas* species (2, 7) showed that *A. stellipolaris* LMG 21856 has 33 unique subsystems, including two tripartite ATP-independent periplasmic (TRAP) transporter collection systems that may play an important role in the transport of nutrients into the cell at low temperatures. Analysis of the *A. stellipolaris* LMG 21856 genome resulted in the identification of an open reading frame that encodes a putative 4-hydroxyphenylpyruvate dioxygenase (HPD) (EC 1.13.11.27), which is a key enzyme involved in ochronotic melanin formation via l-tyrosine catabolism (16). A more detailed analysis of this genome and a comparative analysis with other *Alteromonas* species may provide further insights into the mechanisms they use to survive in different environments.

### Nucleotide sequence accession number.

The complete genome sequence of *A. stellipolaris* LMG 21856 (=DSM 15672) has been deposited at DDBJ/EMBL/GenBank under the accession no. CP013120 The version described in this paper is the first version.
